# Female Fertility Outcome Following Bariatric Surgery: Five-Year Follow Up

**DOI:** 10.1007/s11695-025-08047-5

**Published:** 2025-07-09

**Authors:** Mohamed Shehata, Wael Abosena, Mohamed Elghazeery, Ayman El-Dorf, Mohammad Khirallah, Ashraf El Attar

**Affiliations:** Tanta University, Tanta, Egypt

**Keywords:** Metabolic bariatric surgery, Female fertility, Obesity, Weight loss, Pregnancy outcomes

## Abstract

**Background:**

To evaluate fertility, weight loss, improvement in obesity related comorbidities, and pregnancy outcomes over five years following metabolic bariatric surgery (laparoscopic sleeve gastrectomy [LSG] and laparoscopic Roux-en-Y gastric bypass [LRYGB]) in morbidly obese premenopausal women with infertility or planning pregnancy.

**Methods:**

This retrospective cohort study included 79 adult females with morbid obesity who underwent LSG (*n* = 38) or LRYGB (*n* = 41). After 5 years of follow up: we evaluated percentage excess weight loss (%EWL), comorbidities improvement, postoperative complications or mortality, and fertility outcomes. Fertility outcomes were assessed in three subgroups: primary infertility (*n *= 21), secondary infertility (*n* = 8), and pre-marriage patients (*n* = 50).

**Results:**

Both procedures achieved sustained weight loss (5-year %EWL: LSG 63.1 ± 1.5%, LRYGB 65.2 ± 8.1%; p = 0.16). Conception rates were high across all subgroups (primary infertility: LSG 80%, LRYGB 72.7%; secondary infertility: 100% both; pre-marriage: LSG 100%, LRYGB 92.3%). LRYGB demonstrated superior remission of type 2 diabetes (100% vs 66.7%, p = 0.038) and GERD (85.7% vs 40%, p = 0.015). Pregnancy-related complications, including gestational diabetes and hypertension, were minimal and resolved postpartum. No neonatal anomalies were reported. Cesarean section rates were high in both groups (63.1%–65.8%), likely reflecting cultural or provider preferences rather than surgical indication.

**Conclusions:**

Both LSG and LRYGB are effective and safe in improving fertility, achieving significant weight loss, and supporting favorable pregnancy outcomes in morbidly obese women. LRYGB may be preferred for patients with metabolic comorbidities, especially type 2 diabetes and GERD. Metabolic Bariatric surgery should be considered early in reproductive planning for selected patients.

## Introduction

Morbid obesity is a well-recognized contributor to infertility in females worldwide. The presence of menstrual irregularities, hormonal imbalance, ovulatory disorders and chronic anovulation as polycystic ovary syndrome (PCOS), in addition to altered endometrial physiology are blamed for the infertility in such patients [1].

Beyond infertility, morbid obesity exacerbates pregnancy-related complications such as gestational diabetes (GDM), preeclampsia, and fetal macrosomia, particularly in women with preexisting metabolic disorders like type 2 diabetes (T2D) and hypertension [2]. Therefore, the current guidelines recommend that women with morbid obesity should achieve at least a modest amount of weight loss before seeking for pregnancy [3]. While metabolic bariatric surgery is known to improve these comorbidities, its impact on pregnancy outcomes in this high-risk population remains understudied [4].

Long term goals of reducing weight through dietary and medical means are extremely difficult, especially in those with morbid obesity, with evidence that only 15% of obese subjects undergoing medical weight loss interventions maintain an overall reduction at the long term (> 10 years) [5]. This is even far less in the morbidly obese population. Thus, metabolic bariatric surgery may offer a better alternative in achieving a sustained weight loss in such patients. Surgery to lose weight includes a variety of procedures such as Roux-en-Y gastric bypass, sleeve gastrectomy in addition to various other procedures [6].

These procedures offer varying mechanisms of action, effectiveness, and complications. They promise a more sustained weight loss than conventional nonsurgical methods in those with morbid obesity, as well as better outcomes in the morbid obesity-related comorbidities such as type 2 diabetes (T2D), hypertension, metabolic syndrome, hyperlipidemia, sleep apnea, quality of life, as well as fertility [7].

The aim of this retrospective analysis of prospectively recorded data is to compare the 5 years outcomes of laparoscopic sleeve gastrectomy and laparoscopic Roux-en-Y gastric bypass in terms of fertility outcomes, weight loss, safety, and resolution of morbid obesity-related comorbidities.

## Material and Methods

This is a retrospective cohort study based on prospectively collected data, included premenopausal women who underwent metabolic bariatric surgery (laparoscopic sleeve gastrectomy [LSG] or laparoscopic Roux-en-Y gastric bypass [LRYGB]) between January 2017 and December 2018. After approval from the institute's Research Ethics committee, Faculty of Medicine, Tanta University, Tanta, Egypt (Approval Code: 36264PR917/10/24). Pan African Clinical Trial Registry (PACTR202505787902854), we contacted a cohort of 239 females who have been already followed up for 5 years after their weight loss surgery.

These patients were operated upon in the period between January 2017 and December 2018. Only 197 patients responded, an informed consent was taken for updating their data and for using the data for analysis. In our data system, all patients’ data were prospectively recorded, including their obstetric history. Patients were categorized in two groups: 111 patients underwent laparoscopic sleeve gastrectomy (LSG), and 86 patients underwent laparoscopic Roux-en-Y gastric bypass (LRYGB).

### Inclusion Criteria

1. **Premenopausal status**: Confirmed by regular menstrual cycles (21–35 days) or diagnosis of oligo/anovulation (e.g., PCOS) prior to surgery.
2. **Infertility subgroups**:
  o **Primary infertility**: Failure to conceive after ≥ 12 months of unprotected intercourse, without prior pregnancies.
  o **Secondary infertility**: Inability to conceive following a previous pregnancy, despite ≥ 12 months of unprotected intercourse.
  o **Pre-marriage group**: Women who underwent metabolic bariatric surgery but had not yet married, but planned pregnancy soon after, per cultural and familial norms.

All fertility outcomes were defined per international infertility criteria and verified through medical records and structured patient interviews wherever feasible.

3. **Diagnostic criteria for infertility**:
  o Documented ovulatory dysfunction (e.g., irregular cycles, anovulation) or other causes (e.g., tubal patency confirmed by hysterosalpingography, normal semen analysis in partners).
  o Exclusion of non-weight-related infertility causes (e.g., uterine anomalies, severe male factor infertility).

### Exclusion Criteria

- Postmenopausal women (amenorrhea for ≥ 12 months or FSH > 25 IU/L).
- Prior hysterectomy/oophorectomy.
- Active malignancy or severe systemic disease affecting fertility.

Three subgroups of patients were recognized for analysis. Patients with primary goal of conceiving or complaining from either primary or secondary infertility, as well as those who were seeking weight loss in preparation for marriage, were selected from these 197 patients. These subgroups were defined as follows: primary infertility, secondary infertility, and pre-marriage.

This stratification reflects both clinical definitions and sociocultural fertility contexts, allowing exploration of the impact of metabolic bariatric surgery across a spectrum of fertility motivations.

In the 111 LSG respondents, 14 patients (12.6%) suffered from primary or secondary infertility, and 24 patients (21.6%) were at pre-marriage stage, these **38** were selected for fertility analysis together as group **(I)**. In the 86 LRGYB respondents, 15 patients (17.4%) suffered from primary or secondary infertility, and 26 patients (30.2%) were at pre-marriage stage, these **41** were selected for fertility analysis together as **group (II)**. this is shown in Table 1.

**Table 1:** Incidence of infertility and women in pre-marital period in both groups

Patients	LSG [group I]		LRYGB [group II]	
	*n*. (%)	95% CI	*n*. (%)	95% CI
**Primary infertility**	10 (9%)	15.0%−42.0%	11 (12.8%)	15.7%−41.9%
**Secondary infertility**	4 (3.6%)	4.2%−24.1%	4 (4.6%)	3.9%−22.5%
**Pre-marital**	24 (21.6%)	47.3%−76.6%	26 (30.2%)	48.1%−76.4%
**Total**	**38 (34.2%)**	**90.7%−100.0%**	**41 (47.6%)**	**91.4%−100.0%**

*LSG* laparoscopic sleeve gastrectomy, *LRYGB* laparoscopic Roux-en-Y gastric bypass, *CI* confidence interval

All patients were counseled to defer pregnancy for at least 12 to 18 months postoperatively, in accordance with international guidelines, to allow for completion of the rapid weight loss phase and stabilization of nutritional status. However, in some cases, particularly among those with longstanding infertility or sociocultural pressure to conceive early after marriage, earlier pregnancies were reported. Pregnancies within the first postoperative year were monitored closely for nutritional adequacy and maternal–fetal safety throughout. Several patients conceived within 6–14 months postoperatively, earlier than the recommended deferral period.

We acknowledge that the retrospective identification of patients seeking fertility introduces potential selection bias. However, patient reproductive goals were documented in preoperative counseling records and early follow-up visits, minimizing misclassification. The inclusion was based on clear clinical criteria and consistent documentation in the prospectively maintained electronic system.

In the LSG group **(I)**, age at operation ranged between 18–37 years with a mean/SD of 27.43 ± 5.45 years. In the same patients at the LRYGB **group (II)**, age at operation ranged between 19–38 years with a mean/SD of 26.56 ± 5.66 years. Initial BMI ranged between 35–57 kg/m^2^ with a mean/SD of 43.2 ± 8.2 kg/m^2^ in the LSG **group (I)**, versus 35–60 kg/m^2^ in the LRYGB **group (II)**, with a mean/SD of 46.1 ± 11.4 kg/m^2^. No statistical differences were found between groups with regard to their demographics.

The following data were collected and analyzed for all patients in both groups: preoperative presence and type of morbid obesity-related comorbidity, presence of early or late postoperative morbidity with any impact on pregnancy or delivery, yearly percentage excess weight loss (%EWL) and BMI at conception, comorbidity improvement or worsening during pregnancy and after delivery, as well as the fertility outcomes and nutritional needs during pregnancy in the three subgroups.

Within this cohort, preoperative type 2 diabetes (T2D) or hypertension were identified for targeted analysis. Diagnostic criteria for T2D included HbA1c ≥ 6.5% or use of glucose-lowering medications[8]; hypertension was defined as systolic/diastolic blood pressure ≥ 140/90 mmHg or use of antihypertensive drugs[9]. Gestational diabetes (GDM) was diagnosed per IADPSG criteria; gestational hypertension as BP ≥ 140/90 mmHg after 20 weeks without proteinuria[10]. These comorbidities were tracked preoperatively, at conception, and during pregnancy to assess remission, worsening, or new-onset gestational complications.

#### Outcomes

**Primary outcomes** included fertility rates (conception within the follow-up period), pregnancy complications (gestational diabetes, hypertension), and delivery outcomes (mode of delivery, neonatal anomalies). **Secondary outcomes** included weight loss (%EWL), comorbidity improvement, and postoperative complications.

Fertility outcomes were assessed through patient self-report during follow-up interviews and cross-verified against medical records when available. Comorbidity status was determined using standardized diagnostic criteria and tracked throughout the study period.

To minimize the impact of confounding, we applied strict inclusion and exclusion criteria, clearly defined our subgroups, and collected detailed data on preoperative comorbidities and postoperative outcomes. Demographic and clinical characteristics were compared between groups to identify and account for potential confounders. Where appropriate, subgroup analyses were performed to further explore the influence of confounding variables on the observed outcomes.

#### Statistical Analysis

Data were analyzed using IBM® SPSS® Statistics version 20. Continuous variables (e.g., BMI, %EWL) are presented as mean ± standard deviation (SD) and compared using independent samples t-tests or Mann–Whitney U tests (for non-normally distributed data, assessed via Shapiro–Wilk test). Categorical variables (e.g., fertility rates, comorbidity remission) were compared using Pearson’s chi-square test or Fisher’s exact test where expected cell counts were < 5.

Due to the retrospective nature of the study and the limited subgroup sizes, no a priori sample size calculation or power analysis was performed. The analyses were therefore exploratory and descriptive, with p-values interpreted descriptively and without formal adjustment for multiple comparisons. This approach was chosen given the available data and the study’s primary aim to generate hypotheses for future research. While this limits the inferential strength of our findings, it reflects the real-world constraints of studying this patient population.

Where appropriate, **95% confidence intervals (CIs)** were calculated and presented in the tables to indicate the precision of key effect estimates, including %EWL, conception rates, and comorbidity remission. These intervals provide additional context to support interpretation, particularly given the limited sample sizes.

## Results

The incidence of preoperative morbid obesity-related comorbidities in the LSG **group (I)** ranged between 5.3 to 18.4%, while in the LRYGB **group (II)** it ranged between 4.9–17.1%. These included T2D, hypertension, gastroesophageal reflux (GERD), hyperlipidemia, sleep apnea and orthopedic comorbidity, this is shown in Table 2. No statistical differences were found between groups with regard to their incidence of obesity related comorbidities. Both the studied groups showed marked improvement in the preoperative morbid obesity-related comorbidities. However, by the fifth year of follow-up, the remission and improvement in T2D and in GERD symptoms in the LRYGB **group (II)**, (100% and 85.7% respectively) were significantly higher than the LSG **group (I)** (66.7% and 40% respectively) (P = 0.038 and 0.015 respectively). The improvement in hypertension, hyperlipidemia, sleep apnea and orthopedic problems were comparable in both groups as shown in Table 2. During pregnancy, in the LSG **group (I),** one patient (2.6%) had worsening in her glycemic control with increase in oral drugs dosage, one patient (2.6%) suffered gestational diabetes. Also, one patient (2.6%) suffered gestational hypertension. In this group, neither diabetic nor hypertensive complications were recorded during pregnancy or delivery. Glycemic control and blood pressure returned to their recorded base line after delivery in the three patients. In the LRYGB **group (II),** one patient (2.4%) had worsening in the glycemic control during pregnancy and 2 patients (4.8%) suffered gestational hypertension. There were no recorded maternal or neonatal complications (e.g., preterm birth, growth restriction) specifically attributed to diabetes or hypertension during pregnancy or childbirth. However, by the fifth year, the patient with the worsening dosage did not return to the preconception baseline. This is shown in tables 3–5 in the special group analysis.

**Table 2:** Preoperative comorbidities and their improvement in both studied groups

Comorbidity improvement	LSG group (I) Incidence	LSG group (I) Improvement At 5th year	95% CI	LRYGB group (II) Incidence	LRYGB group (II) Improvement At 5th year	95% CI	*P*-Value
T2D	7.9%	**66.7%**	29.2%−100.0%	12.2%	**100%**	47.8%−100.0%	**0.038**
Hypertension	10.5%	100%	39.8%−100.0%	9.8%	100%	39.8%−100.0%	NS
GERD	13.2%	**40%**	11.8%−76.9%	17.1%	**85.7%**	48.7%−97.4%	**0.015**
Hyperlipidemia	18.4%	71.4%	35.9%−91.8%	14.6%	66.7%	30.0%−90.3%	NS
Sleep apnea	10.5%	100%	39.8%−100.0%	4.9%	100%	15.8%−100.0%	NS
Orthopedic	5.3%	100%	15.8%−100.0%	4.9%	100%	15.8%−100.0%	NS

*LSG* laparoscopic sleeve gastrectomy, *LRYGB* laparoscopic Roux-en-Y gastric bypass, *T2D* type 2 diabetes, *GERD* gastro-esophageal reflux disease, *P-Value* Significant at *p* < 0.05, *CI* confidence interval, *NS* not significant

**Table 3:** Fertility outcomes in the primary infertility patients in both groups

Group	LSG Group (I)	LRYGB Group (II)
Number (*n*.)	10	**11**
Completed successful pregnancy [n. (%), 95% CI]	8 (80%) (49.0%−94.3%)	8 (72.7%) (43.4%−90.3%)
Patients needed assisted reproductive techniques (ART) to get pregnant (n.)	-	1
Maternal age at pregnancy (range)	21–30 years	23–33 years
BMI at pregnancy (mean/SD)	32.3 ± 2.3 kg/m^2^ (30.65–33.95)	32.4 ± 3.5 kg/m^2^ (30.05–34.75)
Time after op (range)	6–12 months	7–14 months
Weight gain during pregnancy (mean/SD)	9.4 ± 4.2 kg (5.89–12.91)	8.9 ± 2.3 kg (6.98–10.82)
Gestational hypertension. [n. (%)]	-	-
Worsening diabetes [n. (%), 95% CI]	1 (2.6%) (0.5%−13.5%)	1 (2.4%) (0.4%−12.6%)
Operative related complications (n.)	-	-
Need extra Iron, Ca, B12 [n. (%), 95% CI]	-	3 (37.5%) (13.7%−69.4%)
Abortion [n. (%), 95% CI]	-	1 (12.5%) (1.6%−37.7%)
Cesarean section [n. (%), 95% CI]	7 (87.5%) (52.9%−97.8%)	6 (75%) (40.9%−92.9%)
Gestational age at the time of delivery (mean/SD)	39.2 ± 0.6 weeks (38.70–39.70)	38.9 ± 1.4 weeks (38.63–40.97)
Neonatal weight at the time of delivery (mean/SD)	3.2 ± 0.5 kg (2.78–3.62)	3.1 ± 0.6 kg (2.60–3.60)
Anomalies (n.)	-	-

*LSG* laparoscopic sleeve gastrectomy, *LRYGB* laparoscopic Roux-en-Y gastric bypass, *BMI* body mass index, *CI* confidence interval

**Table 4:** fertility outcomes in the secondary infertility patients in both groups

Group	LSG	LRYGB
Number (n.)	4	4
Completed successful pregnancy [n. (%), 95% CI]	4 (100%) (39.8%−100.0%)	4 (100%) (39.8%−100.0%)
Patients needed Assisted reproductive techniques (ART) to get pregnant (n.)	-	-
Maternal age at pregnancy (range)	27–33 years	28–32 years
BMI at pregnancy (mean/SD)	32.1 ± 2.2 kg/m^2^ (28.60–35.60)	30.4 ± 1.9 kg/m^2^ (27.38–33.42)
Time after op (range)	10–14 months	9–12 months
Weight gain during pregnancy (Mean/SD)	10.7 ± 2.2 kg (7.20–14.20)	11.3 ± 1.1 kg (9.55–13.05)
Gestational hypertension [n. (%)]	-	-
gestational diabetes [n. (%), 95% CI]	1 (2.6%) (0.5%−13.5%)	-
Operative related complications (n.)	-	-
Need extra Iron, Ca, B12 (n.)	-	-
Abortion (n.)	-	-
Cesarean section [n. (%), 95% CI]	2 (50%) (15.0%−85.0%)	3 (75%) (30.1%−95.4%)
Gestational age at the time of delivery (mean/SD)	39.5 ± 0.4 weeks (38.86–40.14)	38.3 ± 0.7 weeks (37.19–39.41)
Neonatal weight at the time of delivery (mean/SD)	3.3 ± 0.4 kg (2.66–3.94)	3.2 ± 0.3. kg (2.72–3.68)
Anomalies (n.)	-	-

*LSG* laparoscopic sleeve gastrectomy, *LRYGB* laparoscopic Roux-en-Y gastric bypass, *BMI* body mass index, *CI* confidence interval

**Table 5:** Fertility outcomes in the pre-marriage patients in both groups

Group	LSG	LRYGB
Number (*n*.)	24	26
Completed successful pregnancy [n. (%), 95% CI]	24 (100%) (85.8%−100.0%)	24 (92.3%) (75.9%−97.9%)
Patients needed Assisted reproductive techniques (ART) to get pregnant [n. (%), 95% CI]	1 (2.6%) (0.7%−20.2%)	2 (.4.8%) (2.1%−24.1%)
Maternal age at pregnancy (range)	19–24 years	19–25 years
BMI at pregnancy (mean/SD)	30.5 ± 3.1 kg/m^2^ (29.19–31.81)	29.7 ± 4.2 kg/m^2^ (28.08–31.32)
Time after op (range)	12–24 months	11–32 months
Weight gain during pregnancy (mean/SD)	10.4 ± 3.2 kg (9.05–11.75)	9.1 ± 2.3 kg (8.17–10.03)
Gestational hypertension [n. (%), 95% CI]	1 (2.6%) (0.5%−13.5%)	2 (4.8%) (1.3%−16.1%)
Worsening/gestational diabetes [n. (%)]	-	-
Operative related complications (n.)	-	-
Need extra Iron, Ca, B12 [n. (%), 95% CI]	6 (25%) (12.0%−44.9%)	7 (26.9%) (13.7%−46.1%)
Abortion [n. (%), 95% CI]	1 (4.2) (0.7%−20.2%)	2 (7.7%) (2.1%−24.1%)
Cesarean section [n. (%), 95% CI]	15 (62.5%) (42.7%−78.8%)	18 (69.2%) (50.0%−83.5%)
Gestational age at the time of delivery (mean/SD)	39.2 ± 1.4 weeks (38.61–39.79)	39.2 ± 0.9 weeks (38.84–39.56)
Neonatal weight at the time of delivery (mean/SD)	3.3 ± 0.3 kg (3.17–3.43)	3.5 ± 0.2 kg (3.42–3.58)
Anomalies (n.)	-	-

*LSG* laparoscopic sleeve gastrectomy, *LRYGB* laparoscopic Roux-en-Y gastric bypass, *BMI* body mass index, *CI* confidence interval

The observed early deterioration in glycemic control, though limited in our series, mirrors concerns raised in younger cohorts where sleeve configuration, such as antral size, may influence the metabolic durability of T2D remission. Notably, adolescent LSG with reduced antrum length demonstrated superior early T2D remission rates without compromising GLP-1 response or safety[11].

Looking at the impact of early complications on the patients’ fertility or pregnancy [As shown in Table 6], in the LSG **group (I)**, 4 of 38 patients (10.5%) suffered a degree of mild early complications in the form of wound-site infection or electrical burn at the diathermy insulation pad, none of these problems had a recorded impact at the time of pregnancy or delivery. One patient (2.6%) suffered early leaks, at the gastro-esophageal junction. This was managed by mega-stent application and percutaneous US guided drain. However, no impact of this leaks was reported at the time of conception or delivery. In the LRYGB **group (II)**, the 3 patients (7.3%) who complained from minor complications at the time of surgery, two with wound-site infections and one with large hematoma at the site of low molecular weight heparin injection showed no recorded impact at time of pregnancy or delivery. One patient in this group (2.4%) suffered bleeding at the jejuno-jejunal anastomotic site, that necessitated re-laparoscopy and refashioning of the anastomotic site. No recorded impact or complication at time of pregnancy or delivery was reported by this complication.

**Table 6:** Incidence of postoperative complications in both groups

Group	LSG group (I) n. (%) (95% CI)	LRYGB group (II) n. (%) (95% CI)
Minor complications	4 (10.5%) (4.2%−24.1%)	3 (7.3%) (2.5%−19.4%)
Major complications	1 (2.6%) (0.5%−13.5%)	1 (2.4%) (0.4%−12.6%)
Re-intervention	-	1 (2.4%) (0.4%−12.6%)
Significant at *p* < 0.05	***P* = 0.31**	

*LSG* laparoscopic sleeve gastrectomy, *LRYGB* laparoscopic Roux-en-Y gastric bypass, *CI* confidence interval

During pregnancy, one patient in the LRYGB **group (II),** suffered intussusception by the 29th gestational week, presenting by acute abdomen and vomiting, ultrasonography detected intussusception at the jejunojejunostomy junction. The patient was explored laparoscopically and the intussusception was reduced with resection of the ischemic part and refashioning of the alimentary limb, biliary limb anastomosis. This patient had an uncomplicated caesarian section at the 39th week with a full-term healthy baby.

Both groups achieved acceptable weight loss at the first year of follow up, Percentage Excess Weight loss (% EWL) of 67.2 ± 1.3% and 69.5 ± 6.3% in LSG **group (I)** and LRYGB **group (II)** respectively. Percentage Excess Weight loss was maximum by the second year in both groups; 74.1 ± 2.2% and 73.3 ± 2.3% in LSG **group (I)** and LRYGB **group (II)** respectively. By the fifth year, both groups sustained weight loss exceeding 50% of the excess weight loss. 63.1 ± 1.5%, and 65.2 ± 8.1% in LSG **group (I)** and LRYGB **group (II)** respectively. This is lower than our average %EWL per year; 1 st year 72.3 ± 4.2% and 71.6 ± 8.1% and 5th year 67.4 ± 4.1 and 69.2 ± 3.3% in LSG and LRYGB patients respectively. This is probably due to the effect of added gestational weight to the overall results. The overall mean weight gain during pregnancy in our groups was 10.1 ± 3.8 kg and 9.8 ± 2.4 kg in LSG **group (I)** and LRYGB **Group (II)** respectively. The difference between the studied groups was not statistically significant, as shown in Table 7. Individual subgroups weight gain is shown in their tables 3–5.

**Table 7:** Mean percentage excess weight loss at both groups

Mean % EWL	LSG Group (I) (95% CI)	Our LSG overall mean	LRYGB Group (II) (95% CI)	Our LRYGB overall mean
1 st year	67.2 ± 1.3% (66.77–67.63)	72.3 ± 4.2%	69.5 ± 6.3% (67.51–71.49)	71.6 ± 8.1%
2nd year	74.1 ± 2.2% (73.38–74.82)	82.1 ± 3.3%	73.3 ± 2.3% (72.57–74.03)	7.29 ± 5.2%
3rd year	68.1 ± 6.4% (66.00–70.20)	74.2 ± 3.1%	68.4 ± 2.5% (67.61–69.19)	73.4 ± 2.1%
4th year	64.2 ± 2.3% (63.44–64.96)	68.5 ± 1.2%	67.1 ± 1.6% (66.59–67.61)	68.2 ± 5.4%
5th year	**63.1 ± 1.5% (62.61–63.59)**	**67.4** ± 4.1%	**65.2 ± 8.1% (62.64–67.76)**	**69.3** ± 3.3%
P-Value	**P = 0.16**			

*EWL* excess weight loss, *LSG* laparoscopic sleeve gastrectomy, *LRYGB* laparoscopic Roux-en-Y gastric bypass, *P-Value* Significant at *p *< 0.05, *CI* confidence interval

The fertility outcomes in the **primary infertility** patients in both groups are shown in Table 3, it shows that 80% and 72.7% of the primary infertile patients in the LSG **group (I)** and the LRYGB **group (II)** managed to get pregnant. One patient in the LRYGB **group (II)** had a missed abortion and needed later medical assistance in the form of ovulation induction using clomiphene citrate tablets in a dose of 50 −150 mg. This patient got pregnant by the third cycle. The time these patients got pregnant after operation was quite earlier than they were instructed. Despite counseling to defer pregnancy for 12–18 months postoperatively, several patients conceived within 6–14 months postoperatively, earlier than the recommended deferral period. Pregnancies within the first postoperative year were closely monitored due to nutritional risks. Several patients conceived within 6–14 months postoperatively, earlier than the recommended deferral period.

The time passed after operation ranged between 6–12 months in the LSG **group (I)** versus 7–14 months in the LRYGB **group (II)**. Therefore, at time of conception, the mean BMI was still more than 30 kg/m^2^, 32.3 ± 2.3 and 32.4 ± 3.5 kg/m^2^ in the LSG **group (I)** and the LRYGB **group (II)** respectively. In each group, one patient had worsening of her glycemic control, in the LSG **group (I)**, it returned to its base line after delivery, but not in the LRYGB **group (II).** The patients who completed their pregnancy gave birth at a mean of 39.2 ± 0.6, and 39.8 ± 1.4 gestational weeks, and gave birth to neonates with normal birth weight with a mean of 3.2 ± 0.5 and 3.1 ± 0.06 kg in groups **(I)** and **(II)** respectively, with no neonatal anomalies reported. What was striking, was the high cesarean section rates, which were 87.5% in the LSG **group (I)** and 75% in the LRYGB **group (II)**.

In Table 4, the fertility outcomes in the secondary infertility patients in the studied groups are shown. All patients in both groups manage to conceive. No abortions occurred nor need for assisted reproductive techniques (ART) was recorded in these patients. These patients, also, sought pregnancy earlier than advised. The time passed until pregnancy after surgery ranged between 10–14 months in the LSG **group (I)** versus 9–12 months in the LRYGB **group (II)**. At time of conception, the mean BMI was 32.1 ± 2.2 kg/m^2^ and 30.4 ± 1.9 kg/m^2^ in the LSG **group (I)** and the LRYGB **group (II)** respectively. One patient (2.6%) had gestational diabetes in the LSG **group (I)**, her blood sugar levels returned to its base line after delivery, The patients completed their pregnancy and gave birth at a mean of 39.5 ± 0.4, and 38.3 ± 0.7 gestational weeks, and gave birth to neonates with normal birth weight with a mean of 3.3 ± 0.4 and 3.2 ± 0.3 kg in groups **(I)** and **(II)** respectively, with no neonatal anomalies reported. The cesarean section rates were 50% in the LSG **group (I)** and 75% in the LRYGB **group (II)**.

Regarding the patients for whom operative interference was done in the premarital period, high pregnancy rates were observed in both groups: 100% and 92.3% in the LSG **group (I)** and the LRYGB **group (II)** respectively. In these patients, the time passed from surgery to getting pregnant was quite longer, compared to the other patients, ranged between 12–24 months in the LSG **group (I)** versus 11–32 months in the LRYGB **group (II)**. This is probably due to the time taken for setting the marriage up. At time of conception, the mean BMI was 30.5 ± 3.1 and 29.7 ± 4.2 kg/m^2^ in the LSG **group (I)** and the LRYGB **group (II)** respectively. One patient (2.6%) in the LSG **group (I)** and two (4.8%) in the LRYGB **group (II)** needed medical assistance to get pregnant, namely ovulation induction using clomiphene citrate tablets, one patient got pregnant by the third cycle of ovulation induction and the others after four cycles. The incidence of gestational hypertension in these groups were 2.6% and 4.8%, in the LSG **group (I)** and LRYGB **group (II)** respectively**.** All returned to their base line blood pressure after delivery. The gestational age and neonatal birth weight at time of delivery were 39.2 ± 1.4, 39.2 ± 0.9 weeks, and 3.3 ± 0.3, 3.5 ± 0.2 kg, in groups **(I)** and **(II)** respectively, with no reported neonatal anomalies. Here, also, we met high cesarean section rates which were 62.5% in the LSG group **(I)** and 69.2% in the LRYGB group **(II)**. The need for additional supplements in both groups in these patients reflects the rebellion nature of young females refusing adherence to doctor’s instructions, this is shown in Table 5.

No severe maternal/neonatal complications (e.g., ICU admissions, preterm delivery < 34 weeks, or congenital anomalies) were attributed to diabetes/hypertension.

## Discussion

Maternal morbid obesity is an important risk factor in modern obstetrics worldwide. It has been associated not only with higher infertility rates, but also increase in pregnancy associated complications such as gestational diabetes, pre-eclampsia, congenital malformations and fetal growth abnormalities[12]. There is good body of evidence that losing weight can improve reproductive function in women[13]. The mechanism involves normalization of the menstrual cycle and ovulation through restoration of normal hormonal milieu, patients of PCOS showed improvement regarding abnormal hormonal pattern and regaining of spontaneous ovulation[14].

This has been shown also in weight loss after metabolic bariatric surgery which leads to decrease in insulin resistance, and restore the normal levels of both follicular stimulating hormone (FSH) and luteinizing hormone (LH) in patients with PCOS[15]. Therefore, normalization of menstrual cycles after surgery was observed in 71.4% of females with previous menstrual abnormalities[16]. It has also been shown that there are mechanical and psychological changes that improve sexual function and hence fertility[17]. Recent long-term data on adolescent LSG patients further support these benefits, showing sustained weight loss, significant comorbidity resolution, and favorable fertility outcomes, with 76.9% of married participants achieving successful pregnancies over a seven-year follow-up[18].

What is lacking in the literature, is the study of different outcomes in the various bariatric procedures. Most studies looked only into the whole patients’ pool with disregard to those who were attempting to get pregnant[19]. In October 2024, female patients with a minimum of 5 years of follow up from our general pool were contacted. Patients were divided into two groups: 111 patients who underwent laparoscopic sleeve gastrectomy and 86 patients who underwent laparoscopic Roux-en-Y proximal gastric bypass. We looked into fertility outcomes inside these groups in women whose primary goals were seeking pregnancy, whether complaining from primary or secondary infertility as well as those who were seeking marriage. In the LSG patients, 38 were analyzed together as group **(I)**. In the LRGYB, 41 patients were also analyzed together as **group (II) **Table 1.

Metabolic bariatric surgery had shown good safety profile in most studies, with major complications rates ranging from 0.3% to 3%[20]. In our study, the incidence of major complications was 2 (2.5%) patients in total of 79, which is within the accepted incidence. In the LSG group, patient suffered early leak at gastro-esophageal junction, was managed by mega-stent application and percutaneous ultrasound guided drain, which was not followed by pelvic collections or intraperitoneal serious adhesions and did not interfere later with the fertility in this lady who got unaided pregnancy during the study period. In LRYGB patient suffered bleeding at the jejuno-jejunal anastomotic site, that necessitated re-laparoscopy and refashioning of the anastomotic site. The rest were minors with no effect on the studied matter.

Both types of surgery showed similar 5th year % EWL: 63.1 ± 1.5% and 65.2 ± 8.1% in the LSG group and the LRYGB group respectively, the difference was not found to be significant (p = 0.16). In the literature, the 5th year %EWL ranges between 50%−103%[21]. Metabolic bariatric surgery can induce satisfactory sustained weight loss, with improvement in obesity-related comorbidities[22]. It is worth noting that the slightly lower-than-average %EWL may be attributable to early pregnancy-related weight gain. Additionally, this fertility-focused subgroup may differ from the general bariatric population, as women attempting conception may follow different dietary, lifestyle, or supplementation patterns. This selection bias may partly explain the modest variation in weight loss outcomes.

In our study, the remission and improvement in T2D and in GERD symptoms in the LRYGB group (100% and 85.7% respectively) were significantly higher when compared to the LSG group (66.7% and 40% respectively) (P = 0.038 and p = 0.015 respectively). The improvement in hypertension, hyperlipidemia, sleep apnea and orthopedic problems were comparable in both groups.

A primary goal to conceive is high on the list in our female patients; as 34.2% and 47.7% of patients in the LSG group and the LRYGB group respectively expressed. Looking at the fertility outcomes in our two groups: 80% and 72.7% of the primary infertile patients, all patients of secondary infertility, and 100% and 92.3% of pre-marriage patients, in the LSG group and the LRYGB group respectively, manage to get pregnant. In the literature, the overall incidence ranges between 45–54%[23]. In our study, both groups were similar in their fertility rates, the generally increased rates observed in the secondary infertility and pre-marriage patients seems logical, as what they needed mainly was physiological improvements in the pattern of ovulation, this occurs with weight loss[24]. It has been even showed in the literature that the need of fertility treatment was low in these patients[25]. Most of our studied patients, surprisingly, got conceived unaided, especially in the secondary infertility patients. One case in the primary infertility and 3 cases in the pre-marriage patients, one in the LSG group and 2 in the LRYGB group.

The time they took from surgery until they got pregnant showed different patterns in regards to the type of infertility, rather than the type of operation performed. Although current guidelines suggest delaying pregnancy for 12–18 months post-surgery to avoid risks associated with rapid weight loss and nutritional deficiencies, many patients in our cohort conceived earlier (6–14 months). These early conceptions were driven by personal or cultural urgency, particularly in previously infertile or pre-marital patients. No significant complications were observed, but we strongly advise adherence to guideline-recommended timing whenever feasible.

Despite deviation from the 12–18 month guideline in several cases, close monitoring and favorable outcomes suggest a nuanced approach may be warranted in highly motivated fertility-seeking patients. However, standard deferral remains advisable to minimize nutritional risk.

In the primary and secondary infertility patients in both groups, patients got pregnant quite earlier than they were instructed (6–12 months in the LSG group versus 7–14 months in the LRYGB group and 10–14 months in the LSG group versus 9–12 months in the LRYGB group in primary and secondary infertility respectively). Therefore, at time of conception, the mean BMI was still more than 30 kg/m^2^; (32.3 ± 2.3 and 32.4 ± 3.5 kg/m^2^ in the LSG group and the LRYGB group and 32.1 ± 2.2 and 30.4 ± 1.9 kg/m^2^ in the LSG group and the LRYGB group in primary and secondary infertility patients respectively). We generally recommend at least one year of waiting, during the rapid weight loss phase, to maximize the chances of conception and to minimize complications related to obesity. It has been shown (though not uniformly agreed) that early pregnancies might be linked with preterm deliveries (50% for less than 12 months and 20% for more than 24 months), and high spontaneous abortion, especially if malabsorption is involved[26]. In our study, one case (12.5%) of abortion in a patient with primary infertility and 3 cases (6%) in the pre-marriage patients (one in LSG group and 2 in the LRYGB group) were met. It is still recommended that patients need to wait a period of 12–24 months before conception trials[27]. However, it is clear here that our infertile patients cannot wait, as they were anxious to receive their new children. It must be remembered too, that the improvement, in sexual mechanical and psychological functions, is another player here as mentioned before[17]. On the other hand, patients in the pre-marriage phase took quite longer time to conceive, when compared to the other infertile patients. The time passed after operation ranged between 12–24 months in the LSG group versus 11–32 months in the LRYGB group. This is probably due to the time taken for setting the marriage up. At time of conception, the mean BMI was 30.5 ± 3.1 and 29.7 ± 4 kg/m^2^ in the LSG group and the LRYGB group respectively. Our cultural preferences in marriage do not favor too thin women, and this is reflected here. No studies on the pre-marriage patients were found in the literature, we think these are good candidates for surgery to improve their fertility as well as their safety during pregnancy.

We recognize that our sample size is limited, and subgroup analyses are inherently underpowered for definitive comparisons. While the fertility rates appear promising, especially in pre-marriage and secondary infertility groups, statistical significance should be interpreted with caution. These findings should be viewed as exploratory and indicative of trends rather than confirmatory. As such, our findings are best viewed as hypothesis-generating, highlighting potential associations that warrant further investigation. Larger, prospective studies with predefined fertility tracking and adequate statistical power are needed to validate the differential impact of metabolic bariatric surgery across diverse reproductive subpopulations.

In this study, no increase in pregnancy-related comorbidities was observed in all the studied groups. No difference was observed in many other studies between bariatric postsurgical patients and normal weight pregnant women[28]. No nutritional deficiencies were observed generally in our patients during pregnancy. However, in the pre-marriage patients, there was need for added supplements more than the usual in both groups during pregnancy, this reflects the rebellion nature of young females, refusing supplementation and adherence to doctor’s instructions before getting pregnant. Although guidelines insist on supplements of multivitamins and iron after metabolic bariatric surgery, however, little evidence exist that metabolic bariatric surgery can result in any added nutritional deficiencies or anemia during pregnancy[28].

Our findings align with prior evidence that metabolic bariatric surgery mitigates obesity-related metabolic dysfunction, but extend this to pregnancy-specific outcomes[29]. The absence of severe complications (e.g., diabetic ketoacidosis, preeclampsia) in our cohort contrasts with rates reported in obese non-surgical pregnancies, suggesting surgery may reduce risks even in high-risk subgroups[30]. However, the persistence of glycemic dysregulation in one LRYGB patient highlights the need for vigilant postpartum monitoring, particularly after malabsorptive procedures.

Gestational age and neonatal weight were adequate in both groups in all studied patients, regardless of the type of infertility. In the literature, those who underwent metabolic bariatric surgery showed no differences in prematurity, low birth weight, recorded macrosomia or neonatal abnormalities when compared to the not obese[31].

In this study, we met high cesarean section rates, which were 62.5% in the LSG group and 69.2% in the LRYGB group in the pre-marriage patients. This was even higher; 87.5% in the LSG group and 75% in the LRYGB group in the primary infertility patients. This is probably an expression of their anxiety towards the precious baby to come. While our CS rates (50–87.5%) exceed typical averages, this likely reflects cultural preference for elective cesarean among first-time or post-infertility mothers, rather than medical necessity. These findings are consistent with regional obstetric trends and should be interpreted in a sociocultural context. In the secondary infertility patients, the cesarean section rates were 50% in the LSG group and 75% in the LRYGB group. These are lower than in other categories of patients, perhaps as these patients had deliveries before, and were not as anxious. Yet, it needs to remember that the cesarean section rates are higher in the metabolic bariatric surgery patients world-wide, rising up to 65.8%[32]. However, this is still lower when compared to the obese pregnant women rates. Ducarme et al.,[33] reported postsurgical cesarean section rates that were nearly half that of obese nonsurgical patients.

While our retrospective cohort study did not employ advanced statistical adjustments for confounding, we minimized bias by confirming no significant baseline differences between the LSG and LRYGB groups in terms of age, BMI, and key comorbidities. Although a randomized controlled trial or propensity score matching would provide stronger causal inference, our study offers valuable real-world insights within the practical constraints of clinical research. The reliability of our dataset was enhanced by the use of prospectively collected clinical records, with recent follow-up conducted to update key outcomes and obtain consent. Although some loss to follow-up occurred, this was transparently reported and remains within the expected range for long-term studies.

Our fertility outcome definitions (including the culturally specific ‘pre-marriage’ category) were consistently applied using internationally recognized clinical criteria, allowing us to capture a unique but clinically relevant patient group. While inclusion of a non-surgical control group or comparison to alternative fertility treatments would have strengthened the design, such approaches were beyond the scope of this observational study. Nevertheless, our findings contribute important preliminary evidence on the reproductive benefits of metabolic bariatric surgery in a challenging and understudied population, and they underscore the need for future prospective, comparative research.

## Limitations

This study has several limitations that must be considered when interpreting the findings. First, the retrospective design and post hoc selection of fertility-seeking patients introduce potential selection bias, particularly favoring those with favorable outcomes. Although fertility intent was documented in many cases, it was not universally captured at baseline, which may limit subgroup validity. Second, the relatively small sample size (*n* = 79) and subdivision into three fertility categories significantly limit statistical power, particularly for detecting rare outcomes or complications. Consequently, results should be viewed as exploratory and hypothesis-generating.

Although demographic and clinical profiles were comparable between groups, unmeasured confounders (such as hormonal profiles, sexual activity, or partner fertility) could not be adjusted for due to the study design. The absence of a non-surgical control group or comparator fertility treatments limits the generalizability of our findings. Additionally, the study reflects a single-center experience in a unique sociocultural context, with high cesarean section rates and early marriage norms, which may not be generalizable to other populations. Fertility and pregnancy outcomes were verified where possible but partially relied on patient self-report, raising the possibility of recall bias.

Finally, no correction for multiple comparisons was applied, and p-values are interpreted descriptively. Although 95% confidence intervals were added for key outcomes to improve interpretability, definitive conclusions require confirmation through prospective, multicenter studies with standardized fertility tracking protocols.

## Conclusions

At five years postoperatively, both laparoscopic sleeve gastrectomy (LSG) and laparoscopic Roux-en-Y gastric bypass (LRYGB) resulted in sustained weight loss, high conception rates, and favorable pregnancy outcomes in women with morbid obesity. LRYGB showed superior remission of type 2 diabetes and gastroesophageal reflux, while fertility outcomes were broadly comparable across procedures. No increase in severe pregnancy-related complications was observed; gestational age and neonatal birth weight were adequate in all subgroups.

### Clinical Implication

These findings suggest that metabolic bariatric surgery can serve as a valuable therapeutic tool in reproductive planning for obese women, particularly when conventional weight loss strategies have failed. However, conception should ideally be deferred until 12–18 months postoperatively, despite sociocultural or personal pressures, to minimize nutritional risks.

### Key Message

LSG and LRYGB are effective and safe options for improving fertility and pregnancy outcomes in obese women, with LRYGB favored for those with significant metabolic comorbidities.

Due to methodological limitations, including retrospective design and small sample size, our results should be interpreted with caution. Future prospective, multicenter studies are warranted to validate these findings and guide clinical decision-making regarding fertility counseling in bariatric surgery candidates.

## Data Availability

No datasets were generated or analysed during the current study.

## Abbreviations

AR: Assisted reproductive technique
BMI: body mass index
EWL: excess weight loss
FSH: follicular stimulating hormone
GERD: gastro-esophageal reflux disease
LH: luteinizing hormone
LRYGB: laparoscopic Roux-en-Y gastric bypass
LSG: laparoscopic sleeve gastrectomy
MBS: metabolic bariatric surgery
PCOS: polycystic ovary syndrome
T2D: type 2 diabetes
